# Tailored support for type 2 diabetes patients with an acute coronary event after discharge from hospital – design and development of a randomised controlled trial

**DOI:** 10.1186/1758-5996-6-5

**Published:** 2014-01-18

**Authors:** Marise J Kasteleyn, Kees J Gorter, Rebecca K Stellato, Mieke Rijken, Giel Nijpels, Guy EHM Rutten

**Affiliations:** 1 Julius Center for Health Sciences and Primary Care, University Medical Center Utrecht, STR 6.131, P.O. box 85500, Utrecht, GA 3508, The Netherlands; 2 NIVEL, Netherlands institute for health services research, Utrecht, The Netherlands; 3 EMGO Institute VU University Medical Center, Amsterdam, The Netherlands

**Keywords:** Type 2 diabetes, Acute Coronary Event (ACE), Distress, Self-management, Self-efficacy, Quality of life, Depression, Primary care, Illness representations

## Abstract

**Background:**

Type 2 diabetes mellitus patients with an acute coronary event (ACE) experience decreased quality of life and increased distress. According to the American Diabetes Association, discharge from the hospital is a time of increased distress for all patients. Tailored support specific to diabetes is scarce in that period. We developed an intervention based on Bandura’s Social Cognitive Theory, Leventhal’s Common Sense Model, and results of focus groups. The aim of this study is to evaluate the effectiveness of the intervention to reduce distress in type 2 diabetes patients who experienced a first ACE.

**Methods:**

Randomised controlled trial. Two hundred patients are recruited in thirteen hospitals. A diabetes nurse visits the patients in the intervention group (*n* = 100) at home within three weeks after discharge from hospital, and again after two weeks and two months. The control group (*n* = 100) receives a consultation by telephone. The primary outcome is diabetes-related distress, measured with the Problem Areas in Diabetes (PAID) questionnaire. Secondary outcomes are well-being, health status, anxiety, depression, HbA1c, blood pressure and lipids. Mediating variables are self-management, self-efficacy and illness representations. Outcomes are measured with questionnaires directly after discharge from hospital and five months later. Biomedical variables are obtained from the records from the primary care physician and the hospital. Differences between groups in change over time are analysed according to the intention-to-treat principle. The Holm-Bonferroni correction is used to adjust for multiplicity.

**Discussion:**

Type 2 diabetes patients who experience a first ACE need tailored support after discharge from the hospital. This trial will provide evidence on the effectiveness of a supportive intervention in reducing distress in these patients.

**Trial registration:**

NCT01801631

## Background

### General background

Type 2 diabetes patients have an increased risk of an acute cardiac event (ACE) [1-3]. Self-management by patients and their families plays a crucial role to prevent complications in type 2 diabetes. Despite the patients’ own efforts on self-management, they may be confronted with an ACE; 19-23% of the ACE patients have a history of diabetes [4-6]. This confrontation may evoke depressive feelings, not only because of the physical problems, but also because they may experience a loss of control and decreased self-efficacy. In addition, self-management may be complicated, since patients also have to cope with the ACE in daily life [7,8]. It is known that type 2 diabetes and cardiac disease are both associated with decreased quality of life (QOL) [9-11]. The combination of the two conditions decreases QOL even more [12-14]. Taken all these factors into account, it seems that self-management support is of high importance in the first period after the occurrence of an ACE [15]. This should address both the individual patient and the partner, since both have to adapt to the new situation. Furthermore, partners influence the way type 2 diabetes patients cope with their condition [16,17].

Cardiac rehabilitation is widely recommended for patients with an ACE. However, the benefit of the rehabilitation program on physical functioning is significantly lower in type 2 diabetes patients than in patients without diabetes [18]. Appointment adherence is lower and attrition is greater in type 2 diabetes patients as well [19].

Altogether, the above mentioned factors may result in increased diabetes-related distress in type 2 diabetes patients after a first ACE. Distress is an important construct covering concerns about disease management, support, emotional burden, and access to care. Loss of control, feelings of failure and lowered self-efficacy may also play a role [20,21]. Distress in type 2 diabetes is associated with several clinically relevant aspects such as medication adherence and glycaemic control [22,23]. Discharge from the hospital might be a period of increased distress. The American Diabetes Association recommends discharge planning that should include 1) medication reconciliation; 2) structured discharge communication; 3) discharge summary transmitted to primary care physician; 4) follow-up visits scheduled with both primary and secondary care providers. Medication adherence and health status, combined with optimal secondary prevention therapy are also important topics to discuss.

Self-management support that address the specific physical and psychological problems of type 2 diabetes patients who had a recent ACE during the period immediately after discharge from hospital is lacking. Therefore, we developed a theory-based tailored intervention for these patients. This paper describes the intervention and the protocol of the randomised controlled trial (RCT) to evaluate the effectiveness on reducing distress.

**Figure 1:**
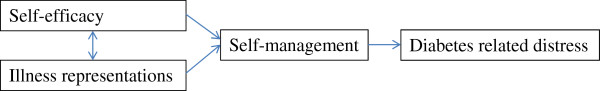
Hypothesis.

We hypothesise that an intervention that succeeds in increasing self-efficacy and improving illness representations will lead to better self-management and decrease diabetes-related distress (Figure 1).

### Theoretical framework of the intervention

Central in our intervention are two psychological concepts that have proven to be key factors of self-management of chronic illness: self-efficacy and illness representations.

#### ***Self-efficacy***

Self-efficacy can be defined as a person’s confidence in his or her ability to perform specific behaviours that are considered to result in beneficial outcomes. According to Bandura’s Social Cognitive Theory, self-efficacy is potentially modifiable and can have an impact on health status, motivation levels and adherence to prescribed regimens [24]. Interventions focusing on improving self-efficacy are successful in improving chronic disease outcomes as well [25,26], such as better self-management in diabetes [27] and cardiac patients [28]. Better self-management can decrease diabetes-related distress without affecting HbA1c [29,30]. A strong relationship exists between self-efficacy and disease-related distress on the one hand and self-management behaviour on the other [31]. Several strategies can be utilised to promote self-efficacy [24,32] such as identifying and reinforcing the patient’s past and present successes or accomplishments of self-management activities.

#### ***Illness representations***

According to Leventhal’s Common Sense Model of self-regulation (CSM), individuals make sense of a health threat by developing their own cognitive and emotional representations of that threat [33]. These representations have an impact on people’s coping behaviour. Cognitive representations include *identity* (beliefs about the condition’s label and associated symptoms), *timeline* (beliefs about the expected duration of the condition), *consequences* (beliefs about the impact of the condition on physical, social and psychological well-being), *curability/controllability* (beliefs about whether the condition can be cured or kept under control through medical treatment and/or self-management behaviour) and *cause* (beliefs about causes of the condition). The *coherenc*e domain concerns beliefs about how well one understands the condition. *Emotional representations* refer to the emotions generated by the condition. Positive illness representations are associated with increased quality of life, better self-management and decreased distress [34,35]. In addition, interventions focusing on illness representations in type 2 diabetes have shown positive effects on well-being and self-management [36].

Self-efficacy and illness representations are related: more self-efficacious persons are more likely to perceive their illnesses to be controllable [37]. This may improve medication adherence [38].

### Focus groups as a basis for the intervention

The intervention is also based on the results of focus groups. Using qualitative methods, we explored the needs and wishes of type 2 diabetes patients and their partners regarding support after an ACE. Participants (*n* = 17, 71% male, aged 61–77) attended the focus groups within six months after discharge from the hospital after their first ACE. The focus groups demonstrated that the patients perceive themselves as a specific group of ACE patients: they have to cope with several diseases at the same time and experience more difficulties after discharge than ACE patients without type 2 diabetes. The participants reported a lack of information supply and missed support from the health care professionals. They had difficulties in coping with physical exercise, sexuality, medication use and the monitoring scheme of the different healthcare professionals. Most patients had no difficulties with nutrition. Participants pointed out that their partners were worried, resulting in overprotective behaviour. Both type 2 diabetes patients and their partners welcomed an individualised self-management support program provided shortly after discharge from hospital. Attention should be paid to the topics described above. Furthermore, the intervention should be tailored to the individual patient’s home environment and involve close relatives.

## Methods

The Medical Research Ethics Committee of the University Medical Center Utrecht has approved the study protocol (Protocol number: 10-403).

### Study design and selection criteria

The study is designed as a randomised controlled trial.

Inclusion criteria:

• History of type 2 diabetes (>1 year)

• Discharged from the hospital after a first acute coronary event defined as a Myocardial Infarction (MI), Coronary Artery Bypass Graft (CABG) procedure or Percutaneous Transluminal Coronary Angioplasty (PTCA)

• Sufficient knowledge of the Dutch language

Exclusion criteria:

• A serious illness or condition preventing full participation

• Inability to fill in questionnaires

### Recruitment

Participants are recruited via their cardiologists in thirteen hospitals, in three different regions across the Netherlands. Within two weeks after discharge from hospital the patients are invited to participate. Patients who agree to participate are asked to provide written informed consent. We use a modified informed consent procedure (Section), to ensure that patients are unaware of the two different conditions [39].

### Modified informed consent procedure

Patients meeting the inclusion criteria will receive the following information in a general information letter:

1) A program has been developed to support patient with type 2 diabetes in the period after discharge from the hospital.

2) We cannot inform you about the exact contents of this program.

3) To evaluate whether the program is effective, we ask you to complete a set of questionnaires twice (directly after discharge and five months later).

4) After your consent you will be informed about your program. You can decide whether you still want to participate.

5) There are no risks associated with participation in this study.

After randomisation participants will be further informed about the program.

### Randomisation

After informed consent, patients are randomised to the intervention or the control group. Randomisation is generated at the patient level by a computerised random-number generator at the research centre.

### The intervention

A trained diabetes nurse (see below), visits the patients in the intervention group at home three times. The first visit (65 minutes) is within three weeks after discharge from the hospital; the second visit (45 minutes) is two weeks later and the third visit (45 minutes) is two months after the second home visit. The number of visits was established based on the results of previous research [40-42] and on feasibility of implementation on a larger scale if the intervention proves effective. During the sessions, the nurse follows a protocol and the patient uses a handbook with assignments and homework. We encourage the patients to invite a partner or close friend who is actively involved in the discussions during the visits.

As a result of the focus group discussions, ten topics were chosen to be discussed during the visits: physical activity, sexuality, pharmacotherapy, the monitoring scheme with different health care professionals, coping together with the partner, coping with both diabetes and the ACE in daily life, (depressive) feelings, nutrition/diet, other.

We incorporate strategies of motivational interviewing in our intervention. Motivational interviewing is a patient-centred counselling approach that actively engages patients in defining experienced problem areas and potential strategies to tackle issues related to illness [43,44]. Interventions based on motivational interviewing show positive effects on patients’ self-management, self-efficacy and quality of life [45].

In the first visit, the focus is on information exchange and strengthening self-efficacy. To explore the individual needs of the patient, the patient is asked to indicate to what extent problems are experienced on the list of ten topics, and which three are the most important. The nurse discusses these three topics in depth, using techniques of motivational interviewing. Next the participant sets goals he/she wants to achieve in the next two weeks. The nurse coaches the patient and helps formulate plans to reach these goals. At the end of the session the patient receives a set of leaflets with more information on ACE and diabetes and the homework for the upcoming two weeks is discussed. The patient is asked to keep daily a log to track strategies for coping with events relating to the topics discussed, and to fill out questions assessing representations of diabetes and the ACE. These questionnaires function as a structuring tool during the second visit, not as an outcome measure.

The focus of the second visit is on strengthening self-efficacy and changing misrepresentations and negative representations of illness. To strengthen self-efficacy, the log is discussed to explore the strategies the participant used to cope with problems. The illness representations questionnaires are discussed, to change misrepresentations and negative representations of diabetes and ACE in order to create more congruent illness representations. The goals formulated during the first visit are evaluated. New goals for the coming two months are formulated, including strategies to reach them. Furthermore, the patient is asked to use now weekly a log to track strategies for coping with difficulties in daily life.

The focus of the third visit is on the future. The log is discussed to enhance self-efficacy. The patient indicates again to what extent problems are experienced on the list of topics from the first visit. The remaining problems are discussed in more detail and the patient is coached to propose strategies to deal with them. The goals formulated during the second visit are evaluated and new goals are formulated, along with strategies to reach them. At the end of the visit uncertainties for the future are discussed.

Nine diabetes nurses experienced with motivational interviewing followed a six hours training for the specific purpose of this study. A GP gave general information about ACE. Patients experiences in the hospital were discussed by a hospital based cardiac nurse and the experiences after discharge were discussed by a type 2 diabetes patient who recently had an ACE. A psychologist taught techniques to strengthen self-efficacy and to intervene on illness representations. The executing researcher discussed the different aspects of the study. To check whether the nurses performed the visits correctly and used the information from the training, we will evaluate the audio recordings of all visits of one of the patients of each nurse. In addition, meetings will be organised twice a year with the nurses and executing researcher to evaluate the nurses’ performance.

The drafted intervention became definite after a pilot study in three patients with three different nurses. Overall, the nurses and patients were positive about the content of the intervention. The intended duration of the visits seemed to be too short and we prolonged the three visits with five minutes each.

### Attention control group

In this study, an attention control group is used. The rationale for using an attention control group (instead of a ‘usual care’ control group) is to distinguish the effectiveness of an intervention tailored to the specific needs of the patient from personal attention without tailored support. In other words: people in both groups receive personal attention, but the intervention group receives tailored support whereas the control group does not. The intervention group receives much more attention than the control group. However designing of an attention control group with a comparable amount of attention is unfeasible. The control group in this study gets significantly more attention with regard to their ACE in relation to their diabetes compared to usual care.

Patients in the control group receive one consultation (approximately 15 minutes) by telephone, within three weeks after discharge from hospital. The aim of the consultation is to give them the opportunity to discuss how they feel and function in the period after discharge; however, no support is provided. Two members of the research team conduct the consultations following a semi-structured protocol. They are instructed to follow the protocol and to give the patient personal attention, without giving any advice or support regarding treatment and self-management. The focus of the consultation is on the topics which are of high importance to the patient. Open questions are used to stimulate the discussion (e.g. “Can you tell me how you have been doing since you got home”).

### Measures

All participating patients receive a set of questionnaires (described in Table 1) at their homes, directly after discharge from hospital (T0) and five months later (T1, one month after the last home visit). The patients’ medical history, medication use and other clinical variables at discharge are obtained from the hospital discharge letter at T0. Clinical variables are obtained with a case report form from the primary care physician at T1.

**Table 1:** Description of questionnaires

**Questionnaire**	**Description**	**Score range**
Problem Areas in Diabetes questionnaire (PAID) [21]	Self-reported questionnaire consisting of twenty statements identified as common negative emotions related to living with diabetes.	Each item is rated on a 5-point Likert scale, ranging from 0 (“not a problem”) to 4 (“a serious problem”). The total score is transformed to a 0–100 scale, with higher score representing higher distress.
WHO-Five Well-being Index (WHO-5) [46]	The five items covering positive mood (good spirits, relaxation), vitality (being active and waking up fresh and rested), and general interests (being interested in things) in the past two weeks.	The degree to which these feelings were present is rated on a 6-point Likert scale, ranging from 0 (“not present”) to 5 (“constantly present”)
		The scores are transformed to a 0–100 scale, with higher score representing better well-being.
Euroqol 5 Dimensions (EQ-5D)/Euroqol Visual Scale (EQ-VAS) [47]	The EQ-5D measures general health status on five dimensions:	The EQ-5D scores was computed using the MVH-A1 algorithm
	1) Mobility	Range -0.594 to + 1.00
	2) Self-care	0: (equal to) death
	3) Usual activities	1: full health
	4) Pain/discomfort	Negative values: a health score worse
	5) Anxiety/depression	than death
	The EQ-VAS measures the overall health state on a graded, vertical line.	Range 0 to 100
		0: worst imaginable health state
		100: best imaginable health state.
Hospital Anxiety and Depression Scale (HADS) [48]	A questionnaire measuring anxiety (7 items) and depression (7 items)	Each item is rated on a 4-point Likert scale, ranging from “Most of the time” to “not at all”.
		Sum scores for each subscale 0–21, higher score indicate more severe anxiety/depression.
International Physical Activity Questionnaire (IPAQ) [49]	29 Items measure how many days’ physical activities are performed during the past seven days in four domains (work, transportation, housework and leisure-time)	Measuring domain-specific activity scores. A total physical activity score is calculated as the sum of the number of minutes of total moderate activity for each subdomain, plus two times the number of minutes of total vigorous for each subdomain.
Active Engagement, Protective Buffering and Overprotection (ABO) [50]	Measuring overprotection by the partner. Five items measure active engagement, eight items measure protective buffering and six items measure overprotection.	Each items is scored on a 5-point Likert scale, ranging from 1 (“never”) to t 5 (“very often”).
		Total score: 15-95
Summary of the Diabetes Self-Care Activities Measure (SDSCA) [51]	Eleven items assessing several aspects of the diabetes regimen: general diet, specific diet, exercise, blood glucose testing, foot care, and smoking. Items measure how many days a patient has performed self-care activities in the last seven days.	Ten items are rated on an 8-point Likert scale, measuring how many days an activity is performed in the last week. One items measures smoking status (yes/no) and the amount of cigarettes smoked in the last week. Each of the domains is measured separately.
Diabetes Coping Measure (DCM) [52]	Four scales measuring diabetes coping: tackling spirit, avoidance, passive resignation and diabetes integration.	The 20 items are measures on a 5-point Likert scale, ranging from 1 (“disagree”) to 5 (“agree strongly”)
		Higher scores on tackling spirit and diabetes integration indicate more adaptive coping. Higher scores on passive resignation and avoidance indicate poor coping.
Confidence in Diabetes Self-care questionnaire (CIDS) [53]	Questionnaire adapted to type 2 diabetes patients. Twenty items measure diabetes-specific self-efficacy.	Each item is scored on a 5-point Likert scale ranging from 1 (“No, I don’t believe I’m able to do this”) to 5 (“Yes, I’m sure I’m able to do this”).
		The total score is transformed to a 0–100 scale, with higher score indicating higher self-efficacy.
Illness Perception Questionnaire (IPQ) – short version [54]	Questionnaire assessing the cognitive representation of illness, focuses on seven scales, assessing (1) *Timeline acute/chronic* and (2) *Timeline cyclical* (3) *Consequences* (4) *Personal control* (5) *Treatment control* (6) *Illness coherence* (7) *Emotional representation*	Eight questions answered on an 11-point Likert scale, ranging from 0 to 10, with the scale for each question having a different meaning. For example when measuring concerns about the illness, the scale ranges from 0 (no at all concerned) to 10 (extremely concerned). The three most important causes of the illness are measured with an open ended question.

The primary outcome in this study is diabetes-related distress, measured with the Problem Areas in Diabetes (PAID) scale. The PAID is a self-reported questionnaire measuring common negative emotions related to living with diabetes and is positively associated with relevant psychosocial measures of distress, including general emotional distress, and negatively associated with self-care behaviour [55]. The questionnaire is well validated and responsive to change in a heterogeneous group of diabetes patients [21,56]. Because distress comprises all important aspects of coping with an ACE for type 2 diabetes patients, the PAID is a good comprehensive primary outcome measure in this study.

Secondary outcomes are well-being, health status, anxiety, depression and clinical variables (HbA1c, blood pressure, cholesterol levels and, body mass index). These variables are mediated by self-management, which in turn is influenced by self-efficacy and illness representations (see Figure 1).

### Sample size

The trial is designed to detect a change in diabetes-related distress as measured by the total score of the PAID. No consensus exists about minimal important differences (MID) of distress measured with this questionnaire. Therefore, we set the MID at half a standard deviation, corresponding to a medium Cohen’s effect size [57]. An analysis of covariance (ANCOVA) will be performed to compare the intervention and control groups while controlling for baseline PAID. Assuming a two-sided significance level of 5%, a power of 80% and a correlation of ρ = 0.3 (“medium” effect size) between baseline en follow-up scores on the PAID, this leads to a sample size of 77 per group [58]. To account for dropout (i.e., no follow-up assessments obtained), we intend to include 100 people in both the intervention and control group.

### Statistical analyses

Data will be analysed according to the intention-to-treat principle, including all patients for whom a follow-up assessment is available. No imputation will be done, as the proposed analysis is already valid under the missing at random assumption [59]. The primary analysis will be an ANCOVA on the change from baseline in total score on the PAID. The model will at least include treatment (intervention or control condition) as factor and the baseline PAID as covariate. ANCOVA has two major advantages compared to a t-test on post-treatment or change scores [60]: 1) ANCOVA correct for baseline score, so possible baseline differences between groups are accounted for without being influenced by regression to the mean; and 2) ANCOVA has a greater power to detect a treatment effect. Secondary outcomes will be analysed similarly. The Holm-Bonferroni correction will be used to adjust for multiple testing [61]. Exploratory sub-group analyses will be performed to assess the impact of age (young vs. old, cut-off: median), gender (man vs. woman), home situation (living alone vs. not living alone) and type of ACE (group 1: MI + CABG or only CABG; group 2: MI + PCI or only PCI; group 3: MI or instable angina pectoris without invasive intervention) on outcomes. Furthermore, a comparison will be performed to investigate whether the improvement of self-efficacy shows the same pattern of improvement as the PAID, by correlating improvement in self-efficacy with improvement in PAID at individual level within treatment groups.

## Discussion

After an acute coronary event diabetic patients have diminished QOL and more diabetes-related distress compared to patients without type 2 diabetes. These patients seem to be in need of additional support in the period after discharge, in addition to the usual care. The American Diabetes Association suggests that specific attention for diabetes is needed when patients are discharged from the hospital after an ACE. The Task Force on Diabetes and Cardiovascular Diseases of the European Society of Cardiology (ESC) and the European Association for the Study of Diabetes (EASD) focus specifically on patients with both diabetes and cardiovascular disease. Since diabetes is associated with increased risk of mortality and complications (including re-infarction or stroke), they emphasise that secondary prevention is of great importance in these patients. According to the guidelines of the ESC and EASD secondary prevention includes altering lifestyle habits, smoking cessation, blocking the renin-angiotensin system, blood pressure control, lipid-lowering medication and blood glucose control [62]. Since secondary prevention comprises altering lifestyle and increased medication use, this can be very burdensome, especially in patients who already had to cope with diabetes. Therefore, self-management support is very important for these patients. Our intervention can help type 2 diabetes patients who experienced an ACE to preform preventive actions and self-management, and reduce distress. Since we use an attention-control group, we may conclude at the end of the study that the results of our intervention are the net result of the content and performance of the planned visits minus just ‘attention’ for the situation a person got in after the discharge from hospital.

In this study, all nurses involved are paid for ten hours per patient, which includes the home visits, training course and evaluative meetings. They will also receive a reimbursement for travel costs. If the current intervention proves to be effective and implementation on a large scale is considered, a large number of nurses could be employed and trained to deliver the additional care, making the intervention less expensive than in this study since they have less travel distances and costs. Whether health insurance companies would pay for the intervention will of course depend on marketing strategies and available resources in due time.

## Competing interests

The authors declare that they do not have competing interests.

## Authors’ contributions

GEHMR is the principle investigator of the trial. The study design and research proposal were worked out by GEHMR, MR, GN and KJG. MJK and KJG are the trial coordinators. MJK drafted the manuscript. RKS was involved in drafting the statistical analysis plan. All authors have corrected draft versions and approved the final manuscript.
